# An overview of systems for providing integrated and comprehensive care for older people in Japan

**DOI:** 10.1186/s13690-023-01076-5

**Published:** 2023-05-04

**Authors:** Junko Sano, Yuzuki Hirazawa, Kohei Komamura, Shohei Okamoto

**Affiliations:** 1 Research Center for Financial Gerontology, Keio University, 2-15-45 Mita, Minato-ku, Tokyo, Japan; 2 Faculty of Economics, Keio University, 2-15-45 Mita, Minato-ku, Tokyo, Tokyo, Japan; 3 Research Team for Social Participation and Healthy Aging, Tokyo Metropolitan Institute for Geriatrics and Gerontology, 35-2 Sakae-cho, Itabashi-ku, Tokyo, Japan

## Abstract

As longevity occurs, people encounter various risks associated with ageing, including economic uncertainty and health issues. Therefore, in addition to extending healthy life expectancy, it is crucial to create an environment where older people can live better even when their intrinsic capacity declines. Additionally, integrated and comprehensive care for older adults is needed to maintain their functional ability and well-being at higher levels. This review provides an overview of the systems and initiatives in Japan, a forerunner of population ageing that supports the quality of life of older people and summarises their remaining challenges. In Japan, with support for access to necessary care available from social welfare councils and community comprehensive support centres, various health and welfare services are provided to respond to the needs of people with different levels of intrinsic capacity, including medical care, preventive care, long-term care, adult guardianship systems, pensions, and social assistance. Nevertheless, there are challenges for the systems, including the gap between life and healthy life expectancy, moderate accumulation and decumulation of retirement assets, lack of human and financial resources for care, and user-unfriendliness and non-covered needs of the current system. Therefore, integrated and comprehensive care beyond health and long-term care is needed to maintain the well-being of older adults, even with their intrinsic capacity declining.

## Population ageing and health

The global population is ageing in developed and developing countries [1]. Japan is one of the most advanced countries regarding population ageing, with 28.9% of the population aged 65 and over in 2021. The percentage is estimated to continue to increase, reaching 38.4% by 2065, with the proportion of people aged 65 or older against the working-age population (i.e. old-age dependency ratio) being 1:3 [2]. Notably, the percentage of the population aged 75 years and over, who may face more health risks, is expected to increase significantly in the future, from 14.9% to 2021 to 25.5% in 2065.

There are two main factors contributing to society’s ageing: an increase in average life expectancy resulting from a decline in the mortality rate and a decrease in fertility rate﻿. The first factor is especially relevant in Japan, which has the longest life expectancy in the world. The average life expectancy in Japan was 81.47 and 87.57 years for men and women in 2021, respectively, and is expected to continue to increase, reaching 84.95 and 91.35 years for men and women by 2065, respectively [2, 3]. As people live longer, they have more chances to encounter various life risks, including health risks and financial uncertainty (e.g. depletion of assets and difficulties in managing assets due to cognitive decline). Although differences exist among individuals, as people age, their intrinsic capacity, defined as ‘the composite of all of the physical and mental capacities that an individual can draw on,’ decreases, and they tend to live with chronic conditions or disabilities [4, 5]. In 2019, the difference between average and healthy life expectancy at birth was approximately 10 years on a global average and approximately 11 years in Japan, implying that people will live for approximately 10 years with some health issues [6].

As previously stated, along with the increase in the percentage of the population aged 75 years and over in Japan, more people may require care and support to respond to their health shocks, considering that the prevalence of dementia and the percentage of people requiring long-term care rapidly increase after the age of 75 [7, 8]. Therefore, in addition to extending healthy life expectancy, creating an environment where people can maintain their well-being is crucial, even with deteriorating physical and psychological health. Thus, achieving universal health coverage, providing appropriate welfare support and health and long-term care services, and creating healthy environments are essential to maintain functional ability, defined as ‘the combination and interaction of intrinsic capacity with the environment a person inhabits’, and the quality of life of older people [4, 5].

Here, we aim to disseminate insights from Japan’s integrated and comprehensive care for older people by summarising the financial and health-related challenges of older people in Japan, physical and psychological changes, difficulties in daily lives associated with ageing, and Japan’s policies to respond to these challenges.

## Health and financial risks among older people

People are more likely to experience various life events, including health and financial issues, as they age. In fact, the most common concern among those aged 60–80 years and older is health issues, followed by economic and financial issues [9]. In Japan, older people tend to have lower incomes and rely on pension benefits for their income [10]. The average annual income per ‘old-age household’ (i.e. a household consisting of persons aged 65 and over only or a household of persons aged 65 and over with unmarried persons aged under 18) was approximately US$25,000, equivalent to approximately 50% of that of households other than old-age household (i.e. ~US$52,000). With the most frequent income category of US$1,100–1,500, accounting for 12.5% of old-age households, their incomes were distributed to lower categories than non-old-age households. Moreover, 62.3% of the average income per old-age household comes from public pension benefits. Despite a decline from 48.4% to 2018, approximately a quarter of old-age households receiving public pension benefits depended solely on their pension benefits as their income in 2020. Furthermore, approximately one-third of them rely on pension benefits for 80–99% of their income, indicating that public pensions play a vital role in older people’s livelihoods.

Contrary to income, older people have more savings on average [11], and the average amount of savings per old-age household in 2019 was approximately US$91,000, about 1.2 times higher than that of non-old-age households (US$77,000). With frequent saving categories of US$150,000–226,000 USD and US$226,000+, accounting for 7.8% and 10.8% of old-age households, respectively, their savings were distributed to higher categories than non-old-age households. The distribution of financial assets was highly concentrated in older households, showing that 63.5% of the total assets in Japan were owned by households with heads aged 60 or older. Therefore, financial assets are also ‘ageing’. Meanwhile, 14.3% of old-age households reported having no savings, indicating considerable economic inequality among old-age households.

Given the life cycle hypothesis [12], older people are assumed to spend more money than their income and meet their living costs by withdrawing assets. However, this does not seem true in Japan [13]. Among households with heads aged 65 or older, the monthly average consumer expenditure and disposable income were approximately US$1,900 and US$2,700 for working households and US$1,500 and US1,600 for non-employed households, respectively. Consistent with this, 51.6% of Japanese aged 60 and over reported that they rarely or never withdraw their savings to cover their daily expenses [14]. Furthermore, this was more remarkable among older groups of those aged 80 or over, with 60.6% and 70.8% for men and women rarely or never withdrawing their savings, respectively. Moreover, even those withdrawing their savings most frequently withdraw just the average monthly amount of US$150–370. A previous study found that non-employed old-age households only decumulated 2.28% of their financial assets, requiring 43.9 years to spend all assets [15].

People may be unable to determine the amount of assets to accumulate and decumulate because of financial uncertainty and health risks. If people live longer than expected, they may face an asset shortfall despite not knowing how long they will live. By living longer, people may have more chances to face health shocks, incurring high medical and long-term care expenses, as a survey shows that approximately 33% of older people spend a large amount on health expenditures [14]. This is also evident from the fact that 64.0% of older adults have some financial concerns related to the aforementioned issues, such as high medical and long-term care expenses (30.8%), admission fees to long-term care facilities (26.0%), and being unable to afford daily expenses (25.8%) [14]. As older people tend to suffer from chronic conditions more often than younger people, large health spending can incur impoverishment. For instance, in Japan, financial hardship arising from the cost of medical and long-term care sometimes leads to the introduction of public assistance [16]. The number of old-age households receiving public assistance has increased annually, and the most common reason for starting to receive public assistance for individuals aged 65 and over is a decrease or loss of savings (49.6%), followed by injury or illness (12.6%) [17]. Thus, impoverishment due to health reasons can be a concern in the context of population ageing.

Notably, even among those with higher incomes, approximately half have financial anxieties. However, unlike their lower-income and savings counterparts, the most common concern is their inability to manage their assets due to dementia or cognitive impairment [14]. With a further increase in the proportion of the older population, particularly those aged 75 years or older, the prevalence of dementia among the whole population may also increase, and estimated to exceed 25% by 2045 [18]. Furthermore, the number of older adults living alone in Japan is projected to increase, reaching 20.8% and 24.5% for men and women by 2040, respectively [19]. This may mean that older adults cannot receive support from their families. Provided that people with dementia increase and large financial assets are owned by them, proper asset management is a vital issue in the context of population ageing.

## Physiological ageing, intrinsic capacity, and potential difficulties experienced in daily life

To respond to the needs of older people, understanding the physical and psychological changes associated with difficulties in their lives, which can occur during normal ageing, is meaningful. The accumulation of molecular damage throughout life causes age-related changes, including frailty, disability, morbidity, and eventually death [4, 20]. With individual heterogeneity, even healthy people undergo various changes in their intrinsic capacity as they age (e.g. sensory system, musculoskeletal and kinaesthetic systems, respiratory and cardiovascular systems, brain and nervous systems, and cognitive functioning) [21–25], they may experience difficulties in their daily life, depending on the degree of these changes.

### Physical and psychological changes

Many elderly people experience difficulties in their daily lives due to changes in their physical and mental functions as they age, although not always to the extent that they require medical or long-term care. For example, in old age, vision and hearing may deteriorate, making it difficult to see distance, read fine prints, hear voices, and safely move around by walking, bicycling, and driving. This may result in increased hazards such as falls and accidents, and may also interfere with interpersonal communication, resulting in a reluctance to go outside or interact with others and social isolation [22, 24, 25]. The musculoskeletal and kinaesthetic systems can also change with age, including a gradual loss of bone mass and density (i.e. osteopenia) and decreases in muscle contractile force, mass, and strength (i.e. sarcopenia) [21, 22, 24]. Combined with declines in other functions (e.g. vision), these changes can induce difficulties in daily activities (e.g. pushing, pulling, and lifting) and decreased ability to walk, balance, and move. [21, 22, 25]. Furthermore, with age, respiratory and cardiovascular functions such as pulmonary function and cardiac function can also decline [21, 25]. With these changes, older people may have difficulty maintaining physical activity for long periods and are more likely to feel fatigued, leading to more sedentary time and refraining from going out.

Additionally, the decline of cognitive function—including intelligence and memory—is a significant change in old age [21, 22, 26, 27]. Age-associated changes in the brain and nervous system result in slower information processing, reflex, and reaction speed [21, 22, 25, 27]. Furthermore, age-related changes include a decline in reasoning and problem-solving ability (i.e. fluid intelligence) [21, 28], and a significant decline in memory—including working and episodic memory—which can lead to more mistakes made by older people, difficulty in multitasking, and forgetfulness [21, 22]. These changes may cause social isolation and difficulties in interpersonal communication, the use of various tools and instruments, and decision-making in daily life. Beyond normal ageing, some older people can undergo pathological ageing and experience multiple chronic conditions, such as morbidity, frailty, and severe dementia. This may worsen mental health by increasing anxiety and depression among older people [22, 24].

In Japan, the rates of patients (per day, per 100,000 population) aged 65 and over in 2020 were higher than any younger ages, with 2,512 for inpatients and 10,044 for outpatient services [29]. Additionally, the number of persons aged 65 and over certified for public preventive or long-term care is 6.69 million, accounting for 18.7% of the insured population [8]. The main factors requiring medical care are cardiovascular diseases (e.g. stroke, hypertension, and heart failure), mental and behavioural disorders, endocrine disorders (e.g. diabetes), musculoskeletal and connective tissue diseases, and gastrointestinal diseases. The main causes of requiring long-term care include cerebrovascular diseases such as stroke, dementia, asthenia due to an advanced age, articular diseases, fractures, and heart disease [29].

### Intrinsic capacity and financial hardships

Financial protection from excessive health expenditure is vital for universal health coverage [30]. However, in the context of population ageing, a decline in the intrinsic capacity may incur different financial difficulties, even without poverty; for instance, they may be unable to go to a bank to obtain cash due to a decline in their physical functions, withdraw their deposits by forgetting the personal identification number, or purchase demanded or needed services and goods due to a complex transaction system (e.g. online shopping). This situation worsens when a person has dementia. Even with mild cognitive impairment, people may live without perceived issues; however, their ability to deal with their finances (e.g. paying bills and managing household finances) and their recognition of these abilities can decline, leading to the purchase of unneeded goods or services, losing money on an investment, and being defrauded [31–33]. Furthermore, several cognitive biases associated with ageing can restrict people from making the optimal choice. First, people may become overconfident in financial decision-making, fail to understand their financial capability, and take considerable or little risk in investment [34, 35]. Second, older people may tend to downplay dangers and threats (i.e. normalcy bias), change their behaviour based on how options are presented (i.e. framing effect), or avoid cognitive loads when facing multiple options by relying more on heuristic processing than younger people [36, 37], which may lead to divergence from a welfare-optimising option. Third, older people can be more loss aversive [38], leading to reluctance to properly decumulate their assets (i.e. endowment effect). In summary, older people may experience financial hardship associated with a decline in their intrinsic capacity, even with physiological ageing, and if they have financial assets.

## Integrated and comprehensive care for older people

**Fig. 1:**
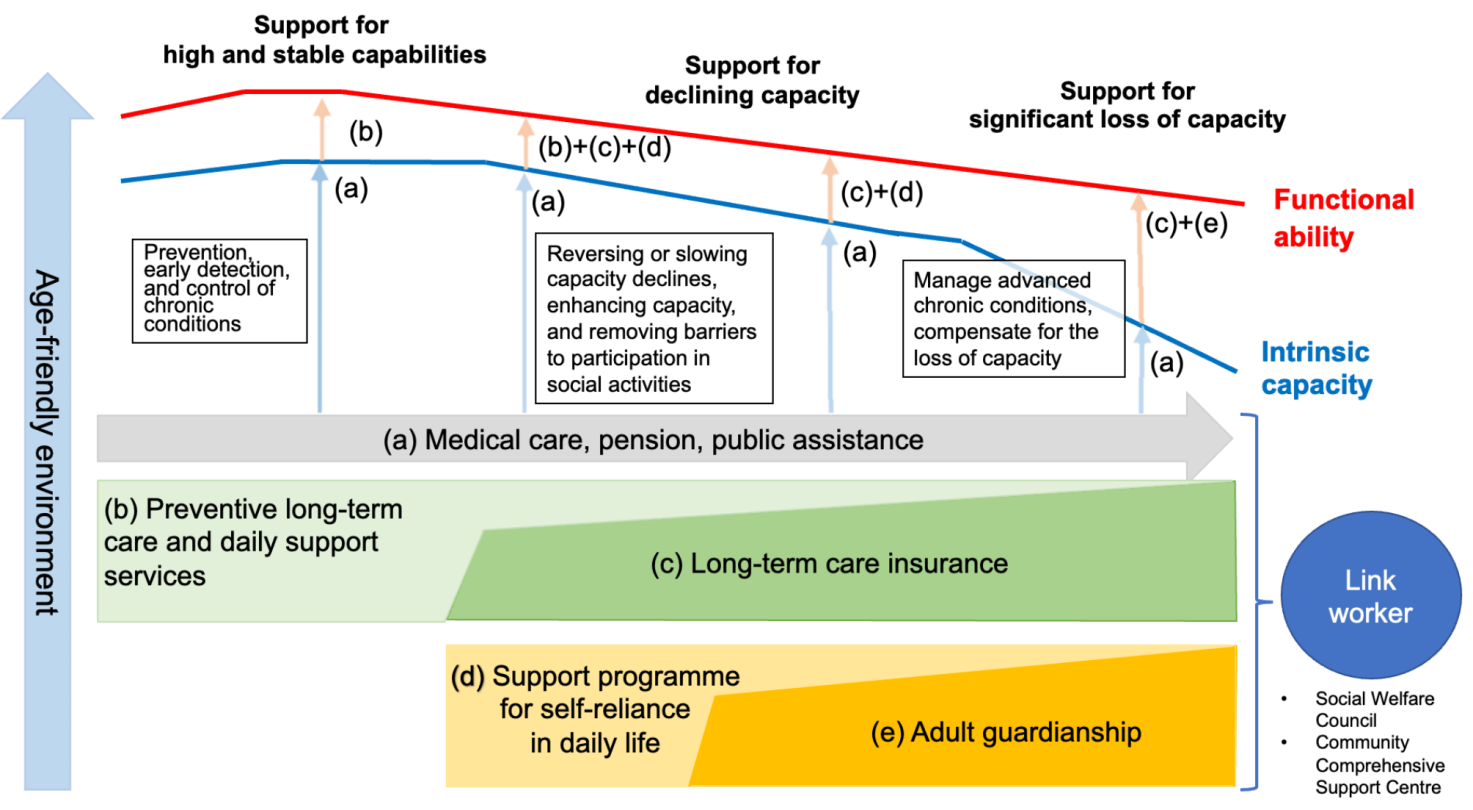
Integrated and comprehensive care for older people in Japan. Source: Created by the authors from [5]; Note: Functional ability means ‘the combination and interaction of intrinsic capacity with the environment a person inhabits’, and the quality of life of older people; Intrinsic capacity means ‘the composite of all the physical and mental capacities that an individual can draw on,’ which decreases with age, and thus some individuals tend to live with chronic conditions or disabilities.

To avoid a further decline in intrinsic capacity or to maintain functional ability and well-being, even if intrinsic capacity declines, providing integrated care for older people (ICOPE) to detect and manage the decline and deliver interventions is essential [5]. Considering that well-being, a good life, or health contains multiple domains and is beyond the mere absence of diseases or disabilities [39, 40], care is required to cover the various needs of older people based on their intrinsic capacity. In alignment with the ICOPE framework, the following four main types of support for older people are available in Japan. Even when one’s level of intrinsic capacity declines after reaching its peak, each support from (a) to (e) in Fig. 1, depending on one’s needs, functions to moderate the decline in functional ability: basic support and systems to support, high and stable abilities, declining capacity, and significant loss of capacity. First, basic support is commonly available for the population regardless of one’s status of intrinsic capacity, including the medical care system, public pension, and public assistance and support for accessing necessary care. The age-friendly environment also helps older people live independently and comfortably regardless of their intrinsic capacity status. Second, a system that supports high and stable capacity aims to prevent, detect, and control chronic conditions, including *Preventive Long-Term Care and Daily Support Services*. Third, the system to support declining capacity focuses on reversing or slowing capacity declines, enhancing capacity, and removing barriers to participation in social activities, including the long-term care insurance system and the *Support Programme for Self-Reliance in Daily Life*. Fourth, the system to compensate for a significant loss of capacity helps people manage advanced chronic conditions, including long-term care insurance and adult guardianship systems. As an individual’s intrinsic capacity is a continuous process, not a dichotomised one (e.g. healthy and unhealthy), these systems provide continuum support based on one’s level of intrinsic capacity and the domains where difficulties occur.

### Basic support

#### Medical care system

There are several basic principles of health insurance in Japan [41]. First, all citizens in Japan are principally mandated to enrol in health insurance (universal health insurance). Health insurance covers various services, such as consultations, treatments, drugs, home-visit nursing, benefits related to hospitalisation (e.g. food expenses), benefits for high-cost medical expenses, dentistry, and a childbirth lump-sum allowance. The services are provided mostly by privately-owned facilities, including local clinics, hospitals, and advanced treatment hospitals (e.g. university hospitals). Second, there is no gatekeeping, meaning that patients can freely choose to visit any medical institution (free access). Even without a referral from a primary care physician, patients can visit a university hospital if they bear a certain amount of costs. Third, fees for services covered by the insurance and their qualities are controlled by the government. Fees for services are uniform throughout the country, and patients can receive the same medical services at the same cost regardless of the level of medical institutions. Fourth, services are provided as in-kind benefits with out-of-pocket payments. Depending on one’s age, the co-payment rate varies from 10 to 30%: 30% for those aged 69 or younger, 20% for those aged 70–74 (30% for those with incomes comparable to the working-age population), and 10% for those aged 75 and over (the same exception as those aged 70–74). The system is financed by these co-payments, contributions from the younger generation, and insurance premiums. Fifth, there are income protection policies. If the co-payment becomes excessive, income protection is provided through tax deductions and reimbursement of costs that exceed the ceiling amount. Additionally, low-income individuals who are not receiving public assistance are eligible for reduction or exemption of insurance premiums and out-of-pocket payments, allowing them to use the services at low cost, based on their ability to pay. Sixth, for early detection and control of chronic conditions, national-specific health check-ups and specific health guidance are available for those aged 40–74 and health check-ups for those aged 75 and older. In each municipality, cancer screening and examinations for sensory organs, such as hearing and vision, and oral health are conducted with the aim of early detection and control of noncommunicable diseases.

#### Universal public pension

The pension system aims to stabilise consumption mainly in old age following the loss of earnings after retirement, reducing uncertainty about the future (e.g. remaining life expectancy after retirement and inflation) [42]. Japan’s public pension system is social insurance managed as a universal pay-as-you-go pension [43]. The pension system has a two-tier structure consisting of a flat-rate national pension and an earnings-related employee pension. Employees’ pensions are primarily for full-time workers, and the premiums are equally shared by an employer and the employed. Additionally, the public pension covers disability and survivors’ pensions to cope with unexpected financial hardship. In principle, those aged 65 years or older with a history of social insurance contributions for 10 years or more are eligible for basic old-age pension benefits (i.e. flat-rate pension). In 2022, the monthly basic old-age pension per person for those with total contributions was approximately US$490 [44]. Employees will receive this basic pension and an employee’s pension based on the length of enrolment and earnings while employed, of which the monthly average is approximately US$1,100.

Several major pension reforms (e.g. 1985 and 2004) have been implemented to improve the pension system’s sustainability, with regular financial verifications every 5 years. With the low fertility rate and ageing society, pension benefits have been suppressed rather than continuously raising premiums for the working-age population. The government has introduced a *macroeconomic slide formula*, which gradually lowers the level of pension benefits per capita in proportion to the progress of population ageing with declining fertility rates. By 2040, the pension level will be lowered by approximately 20%, with an annual reduction of approximately 1%. The pension-eligible age gradually increased from the initial age of 55 to 65 years. Combined with delayed retirement age, pension-eligible age, or the length that people make contributions may be extended in the future.

#### Public assistance

Public assistance is the last safety net to ensure that the entire population can maintain the *minimum levels of wholesome and cultured living* and encourage independent lives [45]. Public assistance is a means-tested benefit targeting relative poverty, requiring continuous assessment by the government regarding the eligibility for public assistance. Approximately 1.64 million households (approximately 2 million people) received public assistance in March 2022, of which old-age households comprised approximately 56% [46]. Among these old-age households, most were single-person households (approximately 52%), potentially because of poverty arising from low assets and pensions. Public assistance provides eight coverages: livelihood, housing, education, medical, long-term care, maternity, occupational, and funeral. Notably, medical assistance has been increasing recently, with households receiving medical assistance from approximately 30% of all recipients [46], partly because older people with chronic conditions needing care rely on public assistance.

#### Link workers: Social Welfare Council and Community Comprehensive Support Centre

Social Welfare Councils and Community Comprehensive Support Centres are located in municipalities throughout Japan to help people access the necessary care. The former are private organisations established with government involvement and play various roles in coordinating care with local governments and civil societies, including matching private providers of welfare services with residents and governments, coordination of stakeholders, role as a federation of welfare organisations and their secretariats, the furtherance of community welfare, and staffing of care workers [47]. Their target population includes older people, children, persons with disabilities, and their respective families. In contrast, the latter focuses mainly on older people with long-term care needs for ageing in place as a community-based integrated care system where people can access continuum care of prevention, medical care, nursing care, housing, and daily life support in a community [48, 49]. To understand citizens’ needs and provide an integrated continuum of healthcare and long-term care, centres are established by each municipality and managed by interprofessional teams of public health nurses, certified social workers, and senior-level long-term care support specialists. Under this scheme, local government officers, healthcare and long-term care providers, and care managers collaborate with the government’s financial support.

### Systems that support high and stable capabilities

#### Preventive long-term care and daily support services

**Fig. 2:**
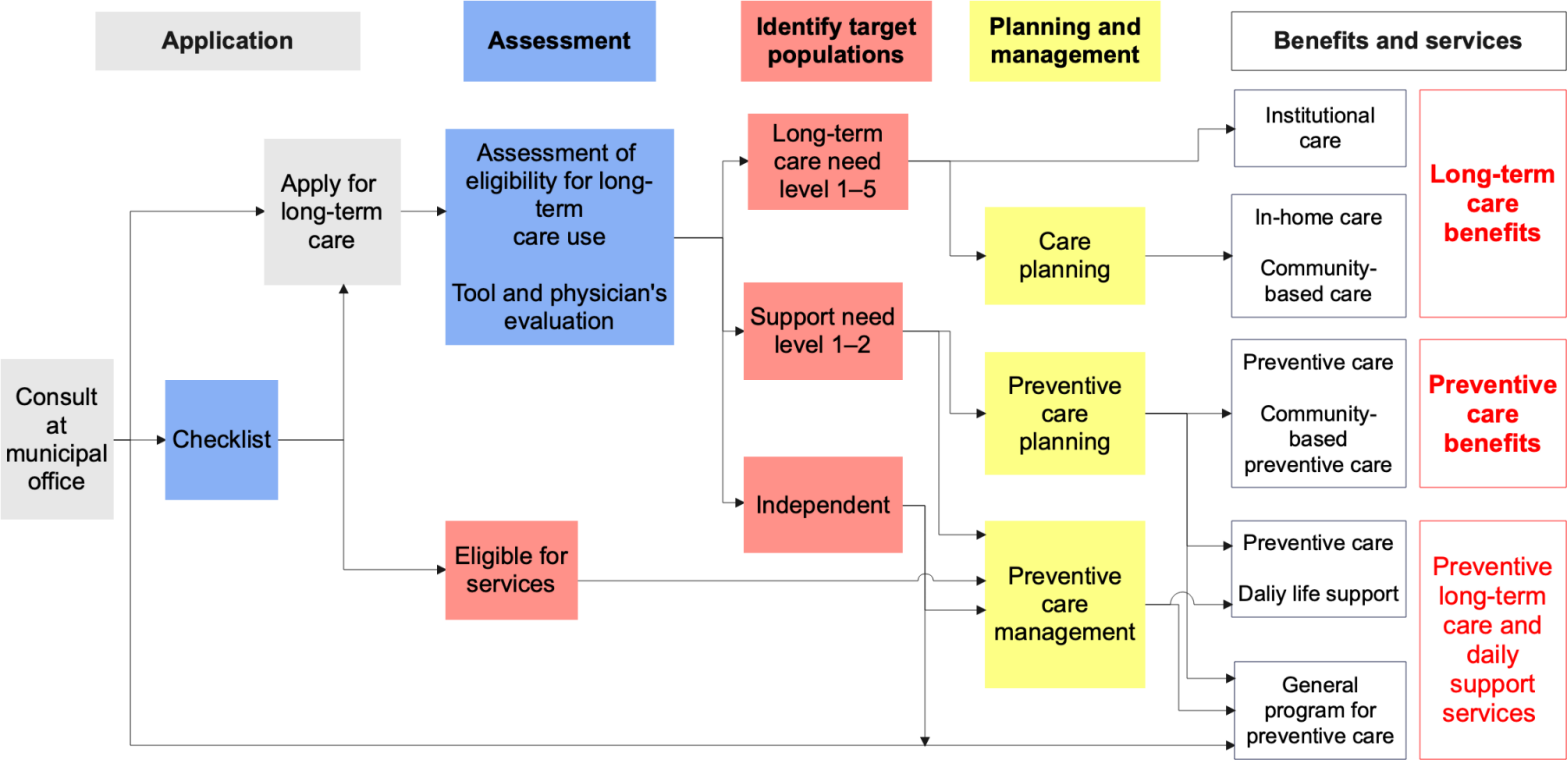
A flow of preventive care and long-term care utilisation in Japan. Source: Created by the authors from [47]

Aiming to create age-friendly communities and encourage social participation of older people to prevent further declines in the intrinsic capacity through the provision of lifestyle support and preventive care by various providers (e.g. volunteers, nonprofits, and private companies), *preventive long-term care and daily support services* was institutionalised in 2015 and enacted in 2017 in all municipalities [50]. These services include holding community salons, watching over and safety confirmation of older people, outing support, support for household chores (e.g. shopping and cleaning), support for caregivers, and support for health promotion and social activities (e.g. employment, hobbies, and volunteer activities). Lifestyle support coordinators have been appointed to develop resources and facilitate efforts in a community by training and finding volunteers and other support providers and networking stakeholders. The target of the services is people aged 65 or older with a mild decline in intrinsic capacity, judged by the long-term care insurance or checklist through their application at the municipal office. The costs of each type of support are determined by each municipality based on services similar to those provided by public long-term care insurance. The eligibility for each service (i.e. daily support, preventive care, and long-term care) is determined by their condition of activities of daily living (Fig. 2).

To enable people to live independently and maintain well-being even with the intrinsic capacity decline, various services should be provided, such as personal care, support for activities of daily living, access to community facilities and public services, financial stability, housing, reducing social isolation and loneliness, and participation in activities that offer life meaning and dignity [5]. These may include services or support that are not covered by public long-term care insurance, such as travel, shopping, and other leisure activities. The use of private goods and services to meet the demands of older adults is also attracting attention [49]. Creating an age-friendly physical environment (e.g. barrier-free and universal design) is crucial in designing goods, services, and living environments to enable older people to participate in various activities.

Through preventive long-term care and daily support services, each municipality, as an insurer of long-term care insurance, is required to support independence and prevent aggravation. Below are examples of initiatives in several municipalities: Toyoake city in Aichi Prefecture has developed services with cooperative societies and private organisations, valuing support to return to a ‘normal life’ for each citizen. Users can request daily life support, including cooking and taking out the trash at an affordable out-of-pocket payment (i.e. approximately US$2 for 30-minute-support). Older people also play a role as support providers; therefore, they can feel a sense of fulfilment and purpose. Additionally, shared rides by private organisations are available for transportation between places where older people may wish to visit (e.g. city halls, clinics, supermarkets, karaoke rooms, and spas, among others) to encourage them to go out. Yasu city in Shiga Prefecture has built a network to monitor consumer safety for the citizens and share information about any abnormal events, working with and sharing the information with various organisations, such as public health centres, clinics, police, financial institutions, retail stores, and civil societies. Obtaining customer lists and other information that the Consumer Affairs Agency confiscates regarding unscrupulous businesses creates a list of residents who may need special attention to protect. Consequently, the city saved potential victims of unscrupulous sales and scams, leading to 811 consumer consultations and a total averted damage of approximately US$140,000 in 2021.

### Systems to support declining capacity

#### Long-term care insurance

To address the growing need for long-term care and reduce the care burden of family members, Japan implemented universal long-term care insurance in 2000, covering all citizens aged 40 and older (see Okamoto and Komamura 2022 for more information on Japan’s long-term care insurance system). Long-term care insurance services can be used according to individual long-term care needs (Fig. 2). Either the person requiring long-term care or their family applies for the service at the municipal office and receives an assessment by the attending physician and an objective tool to determine the level of care required. As a result of the assessment, the patient is classified into three categories: independent, support need level 1–2, and long-term care need level 1–5 (higher need levels represent worse health conditions). Those assessed as independent are eligible for the preventive long-term care and daily support services. For those requiring support need level 1 and 2, preventive care benefits and daily life support are available. In-home care, community-based care, day-care, and institutional care are available for those requiring long-term care need level 1 to 5. When receiving in-home care, the maximum amount of services (fees) covered by insurance each month is determined according to the level of care required, ranging from approximately US$380 for those requiring assistance 1 to approximately US$2,750 for those requiring care 5. Within this range, the co-payment rate is 10% (20% or 30% for those with higher income).

Long-term care insurance is financed by out-of-pocket payments by users, public expenditures, and insurance premiums, principally by all citizens aged 40 years or older. With variations in income and region, the monthly average for people aged 65 or over, whose main source of income is the pension, is approximately US$45 and may further increase in the future. Long-term care services are provided in a quasi-market by contracting with service providers to meet the various needs of users with an application by the people or their family members. However, some people may be unable to do so for some reason (e.g. dementia); to compensate for this, the daily life support project and adult guardianship system, described below, was established as a rights protection system.

#### Support programme for self-reliance in daily life

The Support Programme for Self-Reliance in Daily Life was originally implemented in 1999 to complement the adult guardianship system [51]. The programme aims to support people with an insufficient ability to judge due to cognitive decline, mental retardation, or mental disorder. However, it is granted the capacity to understand the contents of this programme [52]. In this programme, social welfare councils in the community provide support for the independent lives of people. Unlike the certification for long-term care, the criteria are vague; the target population would be people with mild cognitive impairment or other conditions who can perform the procedures to use the service alone. This programme does not cover support for large-value contracts (e.g. real estate transactions), which are covered by the adult guardianship system. However, it provides assistance by support specialists and life support staff in using welfare services, administrative procedures for residence renovation and rental, and day-to-day financial management (e.g. withdrawal of deposit, closing account, and payment of public utility charges, tax, and social insurance premiums), document retention (e.g. personal seals and bankbooks), and life change detection by regular visits. Users pay an average of approximately US$ 10 for services per visit. Only by visiting care services provided by long-term care insurance, some support for financial management is unavailable (e.g. withdrawal of deposit on behalf of the users); therefore, older people may face financial hardship and fail to access their assets. Older people may reach the programme through the support of care managers: approximately 56,000 people used the programme in 2022 [53].

### Systems that compensate for significant capacity losses

#### Adult guardianship system

The adult guardianship system provides legal support to those without sufficient capacity to make decisions because of mental or intellectual disabilities or dementia [54], covering those with more severe cognitive impairment than those with a support programme for self-reliance in daily life. As previously stated, people should apply by themselves and contract with service providers to use long-term care services; however, some people with cognitive impairment may be unable to access these services. Therefore, the system was implemented in 2000 as a set with a long-term care insurance system to support property management (e.g. management of deposits, savings, personal seals, real estate, title deeds, and transactions with financial institutions) and physical custody (e.g. various contracts, including for long-term care, payments, accompanying to clinics to receive a consultation and prescription of drugs, and procedures related to social insurance) of those individuals.

Under this system, legal and voluntary guardians are available. Legal guardians are selected by the domestic court from family members or relatives, legal experts, welfare professionals, welfare organisations, and others to optimise the conservatee’s benefits. In contrast, voluntary guardians are appointed by a person who wishes to use the adult guardianship system if they lose their capacity for judgement. Furthermore, legal guardians are categorised into guardians, curators, and assistants, depending on the capacity to judge of a conservatee. Guardians are allowed to have the authority of representation, whereas curators and assistants are authorised only to perform acts approved by the domestic court to use the person’s remaining capacity. In 2021, most guardians were lawyers, judicial scriveners, and certified social workers, while only 20% were selected from family or relatives [55]. Initially, approximately 9,000 petitions for guardianship in family courts were made in 2000; however, this has increased to approximately 40,000 by 2021, leading to a total of approximately 240,000 users of the adult guardianship system in 2021 [55]. In addition to the adult guardianship system, a private trust is feasible for property management if an agreement with the notarial deed is made well before a person’s capacity for judgement declines. This allows users and their family members to flexibly manage their properties compared to the adult guardianship system; however, this may not be widely used because of its high cost or the absence of a reliable family member for their property management.

## Challenges

In Japan, each system works complementarily to respond to a decline in intrinsic capacity and covers various needs of older people, including medical and long-term care, economic support, and daily life support. Nevertheless, many challenges remain, including those not limited to health, economic, and financial issues, labour force shortages, and challenges of institutions.

### Health

Although Japan is one of the countries with the longest average life expectancy and healthy life expectancy at birth, the gap is approximately 11 years [6], indicating that many people live a long period with some health issues. Therefore, clinical and policy interventions are needed to prevent the decline in the intrinsic capacity extension of healthy life expectancy. With variations by region and insurance type, the average proportion of people receiving specific health checkups in 2020 was approximately 53% overall and approximately 34% among municipal national health insurance [56]. In Japan, noncommunicable diseases account for more than 80% of the disease burden, with large portions arising from neoplasms and cardiovascular diseases [6]. Therefore, prevention, screening, early detection, and control of diseases are essential. Furthermore, addressing the social determinants of health through ‘upstream’ interventions is necessary to reduce the behavioural risks of noncommunicable diseases (e.g. alcohol consumption, tobacco smoking, unhealthy diet, and physical inactivity) [57]. For instance, avoiding adverse circumstances and health conditions during the life course from early to later life is expected to reduce the onset of dementia [26]. Therefore, achieving healthy ageing requires a life-course approach incorporating clinical interventions and interventions for social environments and behavioural risks both from younger to older populations, including promoting social participation among older people, as Japan’s preventive long-term care and daily support services aim. As mentioned earlier, preventive care benefits are available for those with a relatively mild decline in activities of daily living. However, currently, the evaluation of these policies is insufficient. Thus, a scientific evaluation of the effectiveness of these programmes in reducing long-term care need is needed to improve the quality of these services.

### Economic and financial issues

Due to uncertain life expectancy, longevity can generate financial difficulties. As previously stated, most older adults rely on public pensions for their income. However, the proportion of non-regular workers who would only receive benefits from a flat-rate pension has increased. Although older people own more assets than their younger counterparts on average, many of them do not own sufficient assets. These people may experience financial hardship as pension benefits are expected to decline due to the pension system’s sustainability. Therefore, poverty prevention after retirement is important through measures such as improving the treatment of non-regular workers (e.g. extending the application of employees’ pensions) and encouraging asset accumulation. In contrast, some people may have financial difficulties in meeting their needs even if they own sufficient assets due to a lack of financial literacy or a decline in their intrinsic capacity, indicating that education and support for accumulation and decumulation of assets are needed. Therefore, financial advisers who provide neutral advice and create networks in the community for consumer safety are required to protect people with a mild decline in intrinsic capacity who are ineligible for public services.

### Labour force shortage and financial sustainability

Due to the declining birth-rate and population ageing, financing of social insurance—including pension and health and long-term care—is facing financial pressure. As indicated elsewhere [49], the labour force shortage of care workers engaging in long-term care and other services and the financial sustainability of the systems are critical issues. With the necessary number of long-term care workers estimated to increase by approximately 690,000 in 2040 [58], improving the treatment of care workers, utilisation of technologies, and acceptance of foreign long-term care workers are promoted [49]. Reducing those requiring care is also necessary to extend healthy life expectancy and achieve healthy ageing, which may reduce the demands and costs of care and enable extended working life by delaying retirement timing.

A shortage of workers is an issue in covering more older people through the Support Programme for Self-Reliance in Daily Life and the adult guardianship system. Particularly for the adult guardianship system, potential needs would not be fully met if only professional workers (e.g. lawyers, judicial scriveners, and certified social workers) played a central role as guardians. Recently, civil guardians have gathered attention; however, appropriate remuneration is needed because they are principally unpaid, even if high ethics are required.

### Institutional challenges

As previously stated, Japan’s systems for supporting older adults are designed complementarily to provide various services in response to different needs. However, there are challenges for these institutions, such as the complexity of the system and the existence of uncovered areas.

#### Integrated continuum of care

Medical and long-term care are provided by different providers, potentially causing inconvenience and inefficiencies by discontinuous services. Other services we described here do not always have the same entry point and provider. Due to this separation and complexity of the service use, users may have difficulties in accessing necessary care [49]. We previously mentioned that link workers are available to coordinate care. However, link workers and care coordination alone are insufficient to provide people in need with timely care due to limited resources and networks among specialists in different sectors. Community-based integrated health care needs to be further developed to create networks among specialists in different sectors and effectively use limited resources.

#### Programmes for preventive and long-term care insurance

As previously stated, services outside the purview of long-term care insurance coverage are required to enable people to lead independent, meaningful, and dignified lives in the community and maintain high well-being. Thus, preventive care and daily support services in the community emphasise that meeting the various needs of older people by utilising services provided by civil societies and private companies, depending on the situation in each region, is important. Meanwhile, some municipalities may face difficulties in obtaining the necessary labour forces or other resources. Therefore, further consideration is required to achieve successful community care, even in regions with scarce resources.

#### Support programme for self-reliance in daily life and adult guardianship system

Challenges exist, particularly in service accessibility and user-friendliness, for support programmes for self-reliance in daily life and adult guardianship systems, which aim to support the decision-making of people with insufficient capacity for judgement. As previously mentioned, the total number of users of these systems is approximately 300,000 (= 56,000 + 240,000), which would have been significantly higher if people with cognitive decline utilised the services, since the estimated number of people with Alzheimer’s disease and other dementias was 4,000,000 [6]. They may fail to access welfare services without sufficient capacity to make decisions, be unaware of their declining capacity, or be unable to seek help on their own. With an estimated increase in the number of older people living alone, more individuals may need access to care without family support. Approaching these people is essential to ensure access to the necessary care. The support programme for self-reliance in daily life has challenges, such as limited coverage and insufficient complementary relationship between this programme and the adult guardianship system [51, 59]. Although this programme covers only limited services, as previously stated, supporter workers sometimes bear the burden of providing services outside the programme’s purview to respond to users’ urgent needs [59]. Moreover, when users undergo further cognitive decline requiring the adult guardianship system, shifting from this programme to the system may not be seamless [51]. Therefore, flexible programme management, improving supporter workers’ treatment, and creating a mutually complementary relationship between this programme and the adult guardianship system are needed.

The adult guardianship system faces challenges in its coverage. When determining a treatment plan for a conservatee or being hospitalised, a non-relative third-party guardian does not have the right to provide informed consent for treatment or become their guarantee. Furthermore, when a conservatee is hospitalised, they may have to prepare pyjamas, towels, and toiletries. Contracting to rent or purchase these items is a legal act and is included in the duties of a guardian; however, bringing these items from the conservatee’s home is not included. Moreover, since the guardianship ends upon a conservatee’s death, posthumous affairs are not necessarily the guardian’s duties. Even when a relative or family member plays the role of a guardian, issues—such as economic abuse—may occur. For instance, a family member appointed as a guardian and expected to inherit the property may wish to avoid a reduction in their inheritance by underusing the conservatee’s assets for themselves. Thus, to ensure that any system covers necessary support, close coordination between the systems and reflection on a conservatee’s will by understanding their preferences well before needing support is essential. Moreover, fees for services can be a heavy financial burden on users. Once a legal guardian is appointed, it cannot be changed or cancelled until the conservatee passes away. The domestic court determines the guardian’s remuneration based on the person’s assets and duties; the monthly fixed remuneration until death is approximately US$150–450 plus additional payments when dealing with special issues. From the professionals’ perspective, this amount may be small even though the workloads are heavy and continue for a long duration. In March 2022, the Cabinet approved the plan for promoting the use of the adult guardianship system, incorporating guidelines on creating networks for decision-making support among various stakeholders (e.g. public health, medical, welfare, long-term care, and financial organisations), the appointment or alternation of appropriate guardians, appropriate remuneration, and fraud prevention by working with financial institutions, courts, professional organisations, and civil societies [60]. Utilising limited human resources, reducing the financial burden, and appointing an appropriate guardian upon tasks (e.g. switching between a professional or family member) may enhance efficiency.

## Conclusions

To maintain the quality of life of older people, medical and long-term care services to support the decline in activities of daily living and personal, psychological, economic, and social care are essential. In Japan, complementary systems, including financial protection, medical and long-term care, daily life support, and support for financial management and physical custody, enable older adults with a decline in their intrinsic capacity to maintain their functional ability. Nevertheless, many challenges remain, such as the gap between life and healthy life expectancy, accumulation and decumulation of assets, labour force shortage, financial sustainability of the systems, user-unfriendliness of the systems, and uncovered needs. Furthermore, even within Japan, regional heterogeneity exists in population structures and sizes, geographic characteristics, and resources; therefore, the design of community care requires consideration of these differences to enable citizens to age in place and maintain their well-being. Obviously, lessons learned from Japan should be contextualised for a country implementing similar services, considering their demographic, social, economic, geographic, and cultural attributes. Detailed case studies of successes (or failures) of care coordination in different regional settings are useful, enabling policymakers to learn implications from a region with the similar context. Integrated and comprehensive care for older people is needed beyond medical and long-term care to enable them to maintain their functional ability and well-being in response to a decline in their intrinsic capacity.

## Data Availability

Not Applicable.

## Appendix

- Preventive long-term care and daily support services (Kaigo Yobou Nitijou Seikatsu Sien Sougou Jigyou)
- Support programme for self-reliance in daily life (Nitijou Seikatsu Jiritsu Sien Jigyou)
- Adult guardianship systems (Seinen Kouken Seido)
- Social Welfare Council (Syakai Fukushi Kyougikai)
- Community Comprehensive Support Centre (Chiiki Houkatsu Sien Senta-)
- Community-based integrated care system (Chiiki Houkatsu Kea Shisutemu)
